# Preliminary study for non – invasive optical detection of squamous and basal cell carcinomas

**DOI:** 10.1186/1475-925X-11-88

**Published:** 2012-11-26

**Authors:** Ahmed Mohammed Ali, Munqith Saleem Dawood, Mohammed Kadhim Taher, Faeza Aftan Zghair

**Affiliations:** 1 College of Engineering, Medical Engineering Department, Al-Nahrain University, Baghdad, Iraq; 2 College of Medicine, Dermatology Department, Babylon university, Babylon, Iraq

**Keywords:** Skin cancer detection, Diffusion theory, Oblique incidence diffuse reflectance, Reflectometry

## Abstract

**Background:**

The early detection of skin cancer may highly increase the chances of its healing. One of the non-invasive methods of such detection based on the Oblique- Incidence Diffuse Reflectance (OIDR) measurements of the reflected diode laser light from the skin. In this research we designed and implemented the OIDR reflectometry measuring system with a 650 *nm* diode laser source to aid physicians in diagnosing both squamous cell carcinomas (SCC) and basal cell carcinomas(BCC).

**Method:**

The laser is delivered obliquely to the skin surface by an optical fiber fitted through a tube holder of CCD camera. The diffused reflected laser light from the skin is captured by the CCD camera and sent to a computer, which is supplied by a specially prepared Matlab program to analyze these images in order to decide in a time whether the lesion is malignant or benign. Fifty cases were diagnosed under supervision of the consultant section of The Governmental Specialized Marjan Teaching Hospital – MOH – Iraq.

**Result:**

The fifty diagnosed cases by this technique, the results were 90% accurate.

**Conclusion:**

The method of laser oblique-incidence diffuse reflectance (OIDR) combined with using the developed algorithms that have high classification rates may prove useful in the clinic as the process is fast, noninvasive and accurate.

## Introduction

Skin cancer is the most common form of cancers with increasing rate per year especially in fair skin population. Non-melanoma skin cancers account for about half of all cancers and include basal cell carcinomas (BCC) and squamous cell carcinomas (SCC) [1]. “Current diagnostic methods for skin cancers rely on physical examination of lesions in conjunction with skin biopsy, which involves the removal of tissue samples from the body for examination. Biopsy of large lesions often requires substantial tissue removal. Though this protocol for skin lesion diagnosis has been accepted as the golden standard, it is subjective, invasive, time-consuming and painful. Laboratory results for the determination of histopathology of a suspected tumor may generally take several days. Since suspicious areas are identified by visual inspection alone, there are a significant number of false positives that undergo biopsy. Conversely, many malignant lesions can also be overlooked. There is an urgent need for objective criteria that would aid the clinician in evaluating whether biopsy is required” [2].

Now a days there is a growing demand for accurate and fast models to predict the light distribution in biological tissues to deduce their optical properties from the measurable quantities [3]. One of the measurable quantities is the diffuse reflectance, it is a function of the distance between the observation point and the incident point of a laser beam. The diffuse reflectance is defined as the photon probability of re-emission from inside a semi-infinite turbid medium per unit surface area (skin tissue). Measurements of the diffuse reflectance can be used to determine the optical properties of tissue non-invasively [3].

Biological scatterers are primarily cell nuclei and mitochondria, with diameters ranging from 1 *μm* to 8 *μm*. As the laser light wavelength is smaller than these scatterers, therefore the light interaction can be predicted by Mie scattering theory, which is an exact analytical solution of Maxwell’s electromagnetic field equations, but when the scattering particles are much smaller than the wavelength, the light interaction can be predicted by Raleigh scattering theory, which is a limiting case of Mie theory. Scattering coefficient is defined as the probability of photon scattering per unit infinitesimal path length [4].

In this paper we present a design and implementation of a non-invasive, painless and fast method to deduce the optical properties of skin cancerous suspicion lesions based on the application of the oblique incidence diffused reflectance reflectometry (OIDR) as originally conceived by Wang and Jacques [5,6].

The images of the diffused reflectance for both lesion and healthy adjacent skins of the same patient are captured by a CCD camera, these images are then analyzed and processed by a specially written Matlab program v.10 to perform a logical prediction (diagnosis) for the examined lesion for squamous and basal cancerous cells.

## Materials and methods

### Patients studied

SCC and BCC are types of non-melanoma skin cancer that chosen by Research Section of Medical Engineering / College of Engineering / Al-Nahrain University.

Fifty patients are selected under supervision of Dermatology Consultant Section of The Governmental Marjan Teaching Hospital / MOH – Iraq. Dermatologist identify suspicious skin lesion that were going to be biopsied for routine care. Patients were asked to participate in the study and sign on informed consent approved by the Babylon Health Directorate / MOH – Iraq.

The needed data from each patient were collected, analyzed and recorded before physicians removed the lesion and sent it for biopsy. Histopathological diagnoses was performed by the specialized laboratory of Marjan Teaching Hospital and reported within 6 to 7 days to compare with our recorded logic predicted result.

### Oblique incidence diffused reflectance method

The significant changes that happen in malignant cells make it optically differentiable from benign cells due to the enlargement in their cell nuclei and mitochondria sizes, it is important to know that the nuclei and mitochondria are the major scatterers in the cells, therefore the enlargement of their size considered as the important indicator to the presence of cancer cells that cause increase in light scattering [4,7].

**Figure 1:**
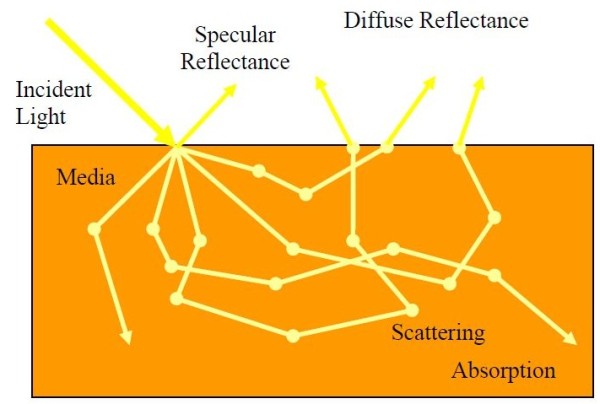
**Light interaction in a scattering and absorbing media **[10] .

When a light enters a semi-infinite tissue, it will generally scatter many times before either being absorbed or escaping the tissue surface at a point other than its point of entry. The multiple scattered light that escapes is called diffuse reflectance [8,9], as seen in Figure 1.

**Figure 2:**
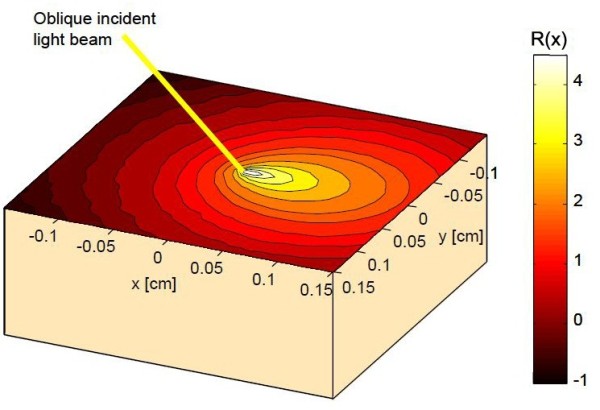
**Single wavelength oblique incidence diffuse reflectance pattern**[10] .

Because it is easier to model isotropic scattering than anisotropic scattering, the reduced or transport scattering coefficient μ_s_' is introduced as the equivalent isotropic scattering coefficient of an anisotropically scattering medium. μ_s_'=μ_s_ (1 – g), where μ_s_ is the scattering coefficient and g is the average cosine of the scattering angle [8,11]:

A sketch for the laser oblique incidence diffuse reflectance pattern from a semi-infinite turbid medium like the biological tissue is shown in Figure 2.

**Figure 3:**
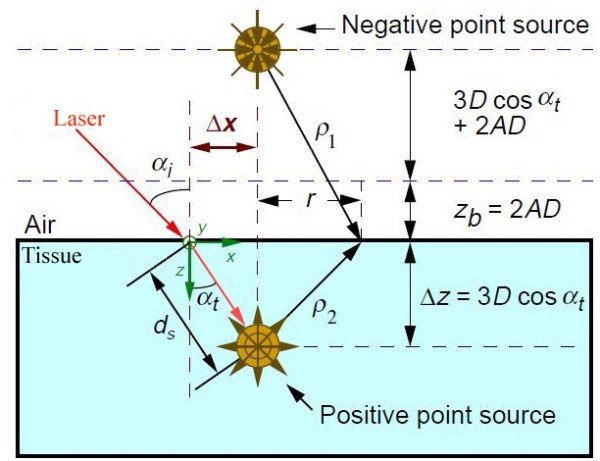
**Schematic representation of obliquely incident light**[6] .

The spatial distribution of diffuse reflectance of an oblique incident laser beam from a semi-infinite turbid medium like the biological tissue has been modeled according to Wang and Jacques by two isotropic point sources; one positive source located below the tissue surface and one negative image source above the tissue surface, as shown in Figure 3. The positive source is buried at distance (*d*_*s*_) from the point of laser incidence on the skin, this distance is considered practically to be three times greater than the diffusion coefficient “D” as in the mean free path Eq.(1) [6,11,12]:

(1) $$1mef=d_{s}=3D=\frac{1}{0.35\mu_{a}+\mu_{s}^{'}}$$

The modified dipole source diffusion theory model gives diffuse reflectance at the skin boundary R(x), by using Eq.(2) which can be scaled to fit a relative reflectance profile that is in absolute units [6].

(2) $$R\left(x\right)=\frac{1}{4\pi }\left[\frac{\Delta z\left(1+\mu_{\mathrm{eff}}\rho_{1}\right)}{\rho_{1}^{3}}+\frac{\left(\Delta z+2z_{b}\right)\left(1+\mu_{\mathrm{eff}}\rho_{2}\right)\exp\left(-\mu_{\mathrm{eff}}\rho_{2}\right)}{\rho_{2}^{3}}\right]$$

Where ;

α_i_ : is the angle between the incident laser beam and the normal line on the tissue surface.

α_t_ : is the angle of light transmission into the tissue, could be calculated according to Snell’s law which is used to measure the new optical path where the isotropic positive point is locate. as seen in Figure 3.

r : is the distance between the normal line between the positive and negative point and the observation point.

*x* : is the distance between the point of observation and the point of light incidence (origin point).

*ρ*_*1*_, *ρ*_*2*_ : are the distances from the two point sources to the point of interest.

z_b_ : is the distance between the virtual boundary and the surface of the tissue.

*A* : is the parameter related to the internal reflection which can be calculated using either Fresnel reflection coefficients or using empirical variable *r*_*i*_ and relative reflection coefficient *n*_*rel*_ of the tissue ambient (air) interface as following [8]:

(3) $$n_{\mathrm{rel}}=n_{\mathrm{tissue}}/n_{\mathrm{ambint}}$$

(4) $$r_{i}=-1.440n_{\mathrm{rel}}^{-2}+0.710n_{\mathrm{rel}}^{-1}+0.668+0.0636n_{\mathrm{rel}}$$

(5) $$A=\frac{1+r_{i}}{1-r_{i}}$$

*A* : is unity for a matched boundary [8].

Δ*z* : is the depth of the positive point source from the surface of skin.

(6) Δz=cosatμa+μs'=Δxtan−1at

**Figure 4:**
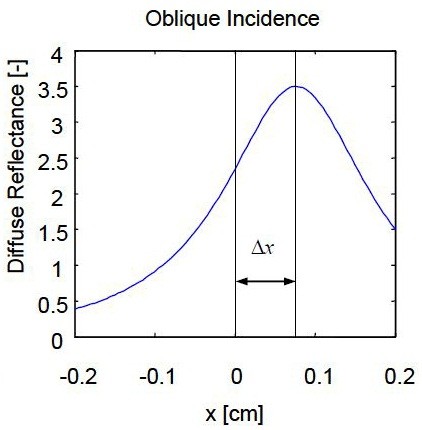
**Oblique incidence diffuse reflectance curve along the x direction**[10] .

Δx : is the distance shift between the point of laser incidence and the center of the most symmetrical circle, as seen in Figures 2, 3 and 4.

(7) $$\Delta x=\frac{sin\;a_{t}}{0.35\mu_{a}+\mu_{s}^{'}}$$

Ones the distance shift (Δx) was found, the diffusion coefficient D could be calculated from Eq.(8) [6]:

(8) $$D=\frac{\Delta x}{3\;sin\left(a_{t}\right)}$$

μ_*eff*_ is the effective attenuation coefficient [6]:

(9) $$\mu_{\mathrm{eff}}=\sqrt{\frac{\mu_{a}}{D}}$$

The μ_*eff*_ value could be found by using a least-square fitting to Eq.(2).

Now it is possible to find the skin optical properties μ_s_' and μ_a_ from Eqs.(10 and 11) as following [6] :

(10) $$\mu_{a}=\frac{\mu_{\mathrm{eff}}^{2}*\Delta x}{3\sin\left(a_{t}\right)}$$

and

(11) $$\mu_{s}^{'}=\frac{\sin\left(a_{t}\right)}{\Delta x}-0.35*\mu_{a}$$

## Experimental work

### Experimental setup

**Figure 5:**
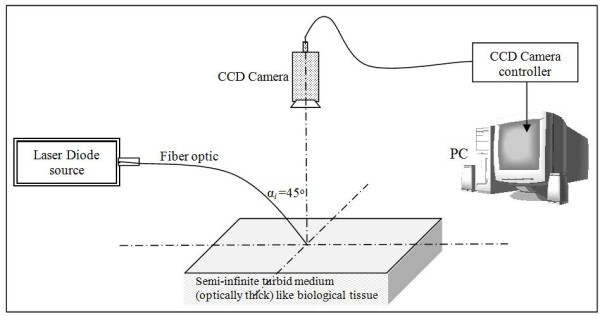
**Schematic sketch of OIDR setup** .

The oblique incidence diffused reflectance reflectometry (OIDR) system that is designed in this work to measure the skin optical properties is sketched in Figure 5. It includes the following components:

1. 650 *nm* laser diode continuous source, DILAS diode laser company.

2. Multimode fiber optic of 0.22 NA and 200 μm in diameter, fitted by a guiding needle at 45^o^ angle with the central normal imaginary line of CCD camera.

3. Charge coupled device (CCD) camera, model F-068D / Delon.

4. Computer with special written analytical Matlab program v.10.

**Figure 6:**
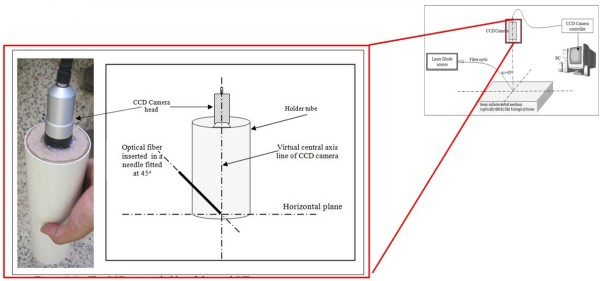
**Geometrical design of CCD camera holder** .

The design of CCD camera holder is shown in Figure 6. The virtual center line (axis) of CCD camera was fitted particularly to be on the center of the horizontal plane, exactly at intersecting point with the needle tip.

**Figure 7:**
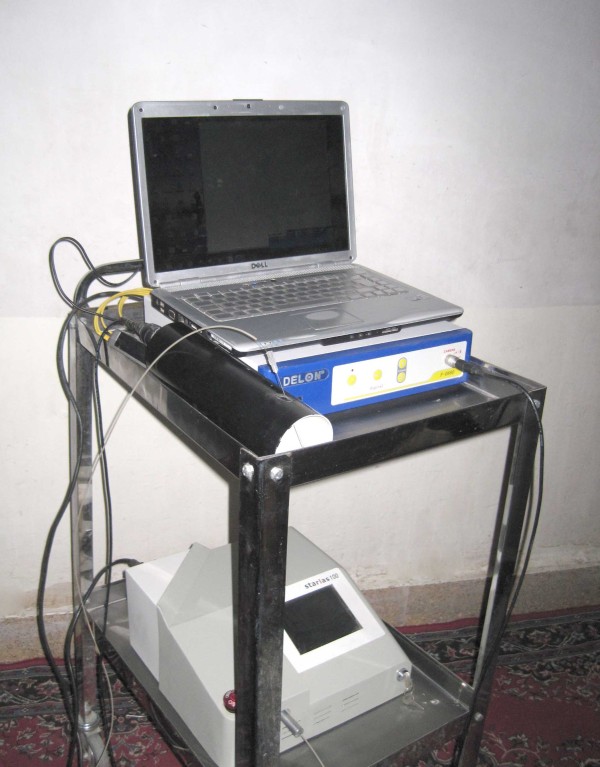
**The implemented OIR laser system for skin cancerous examination** .

The CCD camera holder was painted by black color to reduce the effect of the outside light. The clinic lights were turned off during the examination. All the apparatus were placed on a small portable hospital cart as shown in Figure 7 to move it easily in the patient examination room.

### Work procedure

The lesion and adjacent healthy area were identified and marked by the dermatologist visually with aid of special lenses.

Placing the special holder tube, Figure 6, on the lesion (without pressing) then using the "c" character on the computer keyboard five times to capture five diffused reflected profile images for the suspension lesion while continuously running the laser, this procedure takes about three seconds. Then we repeat this procedure for the adjacent healthy area.

By using "c" character ( capture command ) on the keyboard the computer will collect the raw data from the CCD camera and import it directly to Matlab program directory folder for analysis.

The measurements are repeated five times in order to average the optical properties for each of lesion and healthy skin.

**Figure 8:**
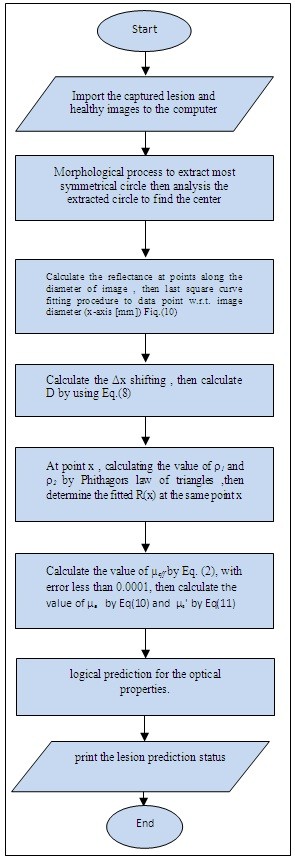
**Work procedure block diagram** .

The optical examination for skin cancer diagnosis was carried on by the sequence shown in Figure 8.

**Figure 9:**
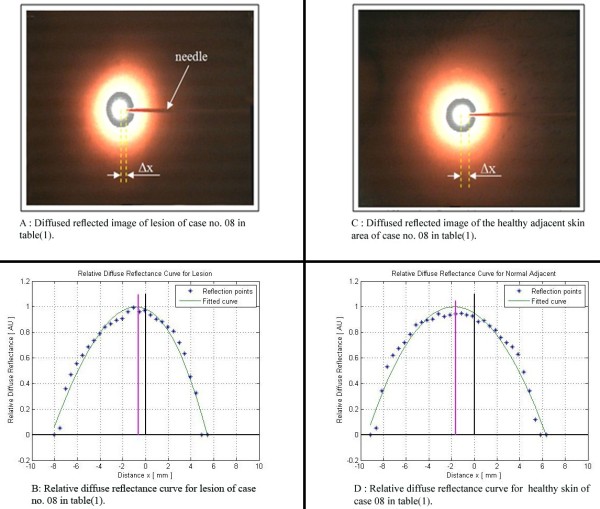
**Diffused reflected images for both lesion and healthy adjacent skin area, with their relative diffuse reflectance curves of case no. 08 in Table**1 .

Figure 9A shows a sample of the diffused reflected image of case no. 08 in Table 1, it was a low grade malignancy cancer, its relative diffused reflectance curve resulted by the Matlab program computations is shown in Figure 9B.

**Table 1:** Results of the 50 cases

**Case No**	**Patient age and Gender**		**Lesion site**	**Skin lesion optical parameters**		**Healthy adjacent skin optical parameters**		**Matlab logical prediction**	**Histological diagnosis (biopsy results )**
	**Gender**	**Age**		**μ**_**s**_**' [cm**^**-1**^**]**	**μ**_**a**_**[cm**^**-1**^**]**	**μ**_**s**_**' [cm**^**-1**^**]**	**μ**_**a**_**[cm**^**-1**^**]**		
1	Female	30	Cheek	6.1033	0.4132	2.5565	0.0013	Low grade Malignancy	SCC
2	Female	45	Breast	4.9191	0.2588	3.0221	0.0011	Low grade Malignancy	SCC
3	Male	60	Cheek	20.1398	0.4108	2.1211	0.0016	Malignant	SCC
4	Male	65	Cheek	40.4127	0.2058	3.1488	0.147	Malignant	BCC
5	Male	53	Cheek	19.3786	0.4267	3.2852	0.1208	Malignant	BCC
6	Male	72	Cheek	13.9541	0.5949	2.0975	0.0016	Malignant	BCC
7	Male	42	Cheek	2.0559	0.0603	2.3434	0.0014	Benign	Benign
8	Male	46	Cheek	5.787	0.3832	3.5257	0.1475	Low grade Malignancy	SCC
9	Male	35	Cheek	5.4704	0.3384	3.6193	0.163	Low grade Malignancy	SCC
10	Male	40	Leg	4.6936	0.2308	3.9528	0.1708	Benign	Benign
11	Male	32	Shoulder	10.0599	0.8058	3.1776	0.1181	Malignant	SCC
12	Male	53	Cheek	12.453	0.857	2.9346	0.2001	Malignant	SCC
13	Male	36	Chest	3.9242	0.8634	2.0341	0.0321	Low grade Malignancy	Benign
14	Male	48	Cheek	7.963	0.1765	2.9361	0.1451	Malignant	SCC
15	Female	47	Cheek	6.5169	0.6583	2.5561	0.1239	Low grade Malignancy	SCC
16	Male	43	Cheek	4.3421	0.6342	2.3692	0.0056	Low grade Malignancy	Benign
17	Male	38	Cheek	7.5231	0.3541	3.4321	0.0239	Malignant	SCC
18	Male	58	Cheek	5.0145	0.8001	3.0189	0.0098	Low grade Malignancy	Benign
19	Male	40	Cheek	12.341	0.4271	3.0341	0.0785	Malignant	BCC
20	Male	46	Cheek	12.4521	0.8765	3.1452	0.0231	Malignant	BCC
21	Male	48	Chest	11.349	0.5023	3.0231	0.1228	Malignant	SCC
22	Female	45	Cheek	10.7432	0.3923	2.9313	0.3817	Malignant	BCC
23	Male	39	Shoulder	3.7213	0.2301	2.9736	0.2001	Benign	Benign
24	Male	51	Cheek	5.8123	0.0969	2.3847	0.0185	Low grade Malignancy	SCC
25	Male	35	Cheek	9.1723	0.7453	2.8376	0.2387	Malignant	SCC
26	Female	46	Cheek	11.3729	0.813	3.0062	0.1078	Malignant	BCC
27	Male	42	Cheek	3.6791	0.2338	2.4312	0.0132	Benign	Benign
28	Male	54	Chest	3.9183	0.5279	2.7128	0.1761	Benign	Benign
29	Female	35	Breast	5.2871	0.2381	3.1829	0.0301	Low grade Malignancy	Benign
30	Female	41	Chest	4.5382	0.2473	3.0031	0.1383	Benign	Benign
31	Male	50	Cheek	8.7812	0.3482	2.4932	0.0762	Malignant	BCC
32	Male	61	Cheek	6.8231	0.4872	3.0045	0.0392	Low grade Malignancy	SCC
33	Male	42	Cheek	7.5237	0.2367	2.4832	0.1289	Malignant	SCC
34	Male	63	Shoulder	12.3287	0.4287	3.2761	0.2313	Malignant	SCC
35	Female	39	Cheek	3.3231	0.1293	2.0213	0.0092	Benign	Benign
36	Male	53	Forearm	10.2382	0.3981	4.2619	0.0312	Malignant	SCC
37	Male	38	Cheek	11.2621	0.2761	3.0123	0.0927	Malignant	BCC
38	Male	43	Cheek	5.9127	0.3327	2.0128	0.0327	Low grade Malignancy	SCC
39	Female	29	Cheek	4.1281	0.0912	2.5321	0.0029	Benign	Benign
40	Female	48	Cheek	6.2632	0.2642	2.9327	0.1234	Low grade Malignancy	SCC
41	Male	45	Cheek	9.4753	0.3424	3.4842	0.2013	Malignant	BCC
42	Male	38	Arm	10.3872	0.2345	2.9478	0.0231	Malignant	SCC
43	Male	56	Cheek	12.3473	0.1283	2.8184	0.0281	Malignant	SCC
44	Male	47	Cheek	18.4732	0.4328	3.045	0.0294	Malignant	BCC
45	Female	34	Chest	6.3424	0.1384	3.3412	0.0634	Low grade Malignancy	SCC
46	Male	44	Leg	13.4721	0.3714	3.1348	0.1392	Malignant	BCC
47	Male	37	Cheek	6.0313	0.3591	3.2467	0.0482	Low grade Malignancy	SCC
48	Female	46	Cheek	4.2485	0.1439	2.3411	0.0927	Low grade Malignancy	Benign
49	Male	33	Cheek	4.4553	0.1943	3.0183	0.0046	Benign	Benign
50	Male	58	Cheek	7.2384	0.3872	2.8274	0.06289	Malignant	SCC

For the same patient case no. 08 in Table 1, Figures 9C and 9D show the diffuse reflected image of the healthy adjacent skin area and its relative diffused reflectance curve resulted by the Matlab program computations respectively.

## Results and discussion

**Figure 10:**
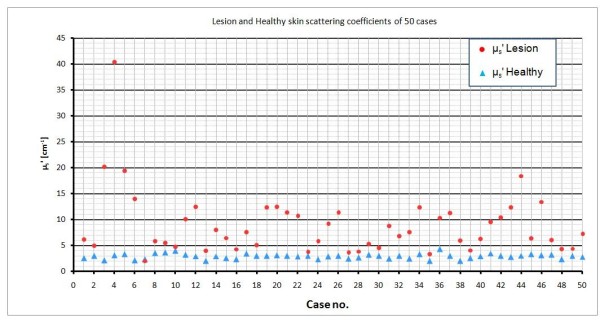
**Lesion and healthy skin scattering coefficients of the all 50 cases** .

**Figure 11:**
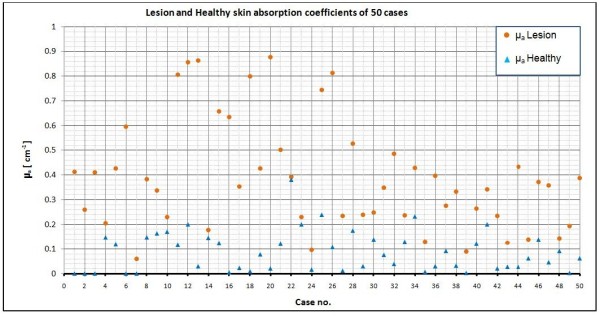
**Lesion and Healthy skin absorption coefficients of the all 50 cases** .

The results are tabulated for fifty examined cases presented in Table 1. Our experimental non-invasive logical prediction decision, beside the invasive biopsy analysis result is shown in the table as well as the optical parameters of the lesion and the healthy skins for each patient. Figures 10 and 11 show the optical properties of the all fifty cases.

This OIDR setup required at least 8 bit of dynamic range of CCD camera to measure the diffuse reflectance within a few centimeters radius of diffused reflectance image, without bring the CCD camera in saturation state.

The accuracy of Δx shift value measurement was proportional to the resolution of the CCD camera in the setup. In our study, the size of pixel was 0.05 mm/pixel and the CCD camera dynamic range was 16 bit.

The sharpness and Δx measurement accuracy of the diffused reflected images, as were shown in Figures 9A and 9C, increased after painting the camera holder tube black.

The 620 – 670 *nm* visible light range is better for these applications than the UV and IR. A red 650 *nm* diode laser was selected for these measurements due to its low absorption in the high scattering epidermal tissue, which increased the accuracy of the measured optical properties. While using a green laser of 532 *nm* wavelength to check this phenomena shows that the reflectance at 532 *nm* was below the sensitivity of the CCD camera system.

**Figure 12:**
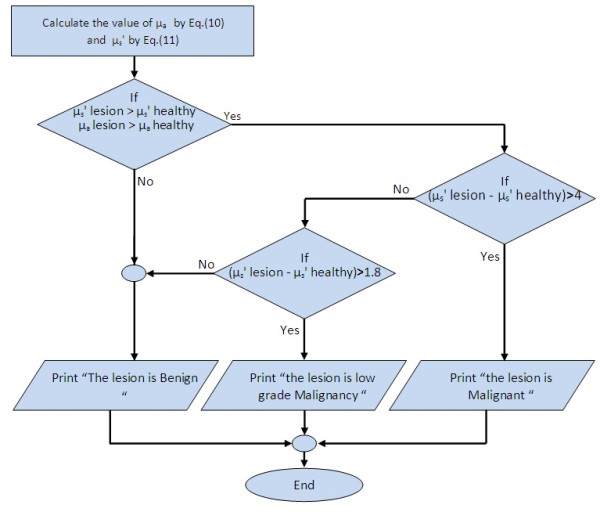
**Decision rule flow chart** .

The diagnostic logical decisions in Table 1 was based on the μ_s_' threshold value selection beside the increment in the μ_a_ value, Figure 12 shows the decision rule flow chart for the 50 patients. If the difference value between the scattering coefficient of lesion and the scattering coefficient of healthy adjacent skin greater than or equal to 1.8 cm^-1^ and less than or equal to 4 cm^-1^ and combined with a small increment in the absorption coefficient of lesion over the absorption coefficient of normal healthy adjacent skin, then this lesion will be considered as a low grade malignancy lesion case.

The threshold value (1.8 cm^-1^) was chosen according to case no. (09) in Table 1, which is of a 35 year male with lesion in the cheek. The biopsy result was low grade malignant and the difference between the reduced scattering coefficient of lesion and adjacent healthy skin was 1.8511 cm^-1^.

While, the threshold value 4 cm^-1^ was chosen according to case no. (15) in Table 1, 47 year female with lesion in the cheek, the biopsy result was low grade malignant and the difference between the reduced scattering coefficient of lesion and normal healthy adjacent skin was 3.9608 cm^-1^.

Therefore, the low grade malignancy threshold considered to be ranged from 1.8 cm^-1^ to 4 cm^-1^ .

If the difference value between the scattering coefficient of lesion and the scattering coefficient of healthy adjacent skin less than 1.8 cm^-1^ and combined with a small increment in the absorption coefficient of lesion over the absorption coefficient of normal healthy adjacent skin, then this lesion will be considered as a benign lesion case.

If the difference value between the scattering coefficient of lesion and the scattering coefficient of healthy adjacent skin greater than 4 cm^-1^ and combined with a small increment in the absorption coefficient of lesion over the absorption coefficient of normal healthy adjacent skin, then this lesion will be considered as a malignant lesion case.

Statistically, there are 36 cancerous patients (based on biopsy result) ware predicted positive by our test, these true positive (TP) patients cases represent 72% of all 50 cases.

While, Five non-cancerous lesion patients (based on biopsy result) ware predicted positive by our test, these *false positive* (FP) patients cases represent 10% of all 50 cases.

Nine non-cancerous lesion patients (based on biopsy result) ware predicted negative by our test, these *true negative* (TN) patients cases represent 18% of all 50 cases.

Finally, all patients predicted negative on our test ware diagnosed negative (based on biopsy result), therefore there is no *false negative* (FN) in our procedure. This was accomplished by choosing the precise (1.8 cm^-1^) threshold value.

Proportion of 9 cases that tested negative (TN) of all the 14 patients that actually are negative (TN+FP) represent *the Specificity* (TNR) of our prediction which it 64.62% (With higher specificity, fewer suspected lesion patients are labeled as cancerous).

While, *the Sensitivity* (TPR) of our prediction results is 100% (its represent the probability that our test is positive given that the patient has a cancer), this was accomplished by having no false negative (FN) cases. Table 2 shows the statistical results.

**Table 2:** Statistical results of the 50 cases

Statistic Item	Result	Notes
True positive (TP)	36	72% of all 50 cases
False positive (FP)	5	10% of all 50 cases
True negative (TN)	9	18% of all 50 cases
False negative (FN)	Zero	0.00% of all 50 cases
Specificity (TNR)	64.62%	= TN/(TN+FP)
Sensitivity (TPR)	100%	= TP/(TP+FN)
The positive prediction value (PPV) the proportion of true positives out of all positive results	87.8%	= TP/(TP+FP)
The Negative prediction value (NPV) the proportion of true positives out of all positive results	100%	= TN/(TN+FN)
Accuracy of our predictive measurements (ACC)	90%	ACC= (TP+TN)/(P+N) Where; P = (TP+FN) N = (FP+TN)

Patients cases no. (13, 16, 18, 29, and 48) in Table 1 were predicted by the Matlab logical prediction as a "low grade malignancy", while the patients histological examination (biopsy result) show negative result for malignancy behavior, these false positive (FP) cases are appeared because the difference value between the reduced scattering coefficient of lesion and normal healthy adjacent skin being close to 1.8 cm^-1^.

**Figure 13:**
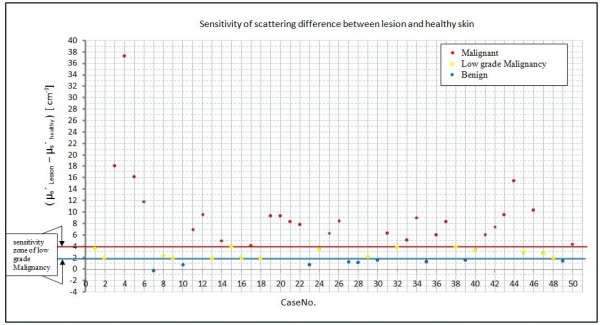
Sensitivity of scattering difference between lesion and healthy skin

Figure 13 shows the results of Matlab logical prediction with two lines of the scattering threshold that represent sensitivity zone of low grade malignancy, the yellow points represent the low grade malignancy cases, the red points represent the malignancy cases, while the blue points represent the benign lesion cases.

Low grade malignancy decision by our system is a critical decision, because it depend on the experience of the physicians to see the value of the difference between the normal and lesion optical properties, as shown in Figure 13. When the difference between normal and healthy of μ_s_' is close to 4 cm^-1^ threshold value, the physician must expect the malignancy behavior of the tested lesion and send the patient to perform a histological examination to be sure about the diagnosis. While, if the difference between normal and healthy of μ_s_' is close to 1.8 cm^-1^ threshold value, the physician must expect the benign behavior of the tested lesion.

The threshold value used to eliminate the false negative (FN) diagnosis, e.g. if we change the threshold (difference) value 1.8 cm^-1^ to be 1.9 cm^-1^, this will produce a false negative diagnosis in case no. (02).

## Conclusions

Only the relative profile of the diffuse reflectance was used for the extraction of optical properties, which made the device insensitive to variations of some system parameters such as intensity of the laser source.

The accuracy of Matlab logical prediction result depends on the image analysis and fitting accuracy to obtain optical properties of lesion and adjacent healthy skins, These results in turns depend on many factors such as the light source wavelength, resolution of CCD camera, and the procedure of estimating the center of the circles and determining the Δx shift, as well as the chosen μ_s_' threshold value.

The overall accuracy of a our predictive measurements is 90% which represent the degree of closeness of our predictive measurements to the actual (true) diagnoses of suspected lesion, see Table 2.

The common site of SCC and BCC is on the face, therefore it was important to measure the accuracy of the method with respect to the lesion site, the accuracy of the measurements was 91.6% for all lesions that are on the cheek, Table 3. The mean value of the reduced scattering coefficient for the healthy cheek skin is 2.8 cm^-1^, that have the statistical variance of 0.1842, and the standard deviation of 0.4292 cm^-1^.

**Table 3:** The lesions that are on the cheek

**No.**	**Case No**	**Patient age and Gender**		**Lesion Site**	**Skin lesion optical parameters**		**Healthy adjacent skin optical parameters**		**Matlab logical prediction**	**Histological diagnosis (biopsy results )**
		**Gender**	**Age**		**μ**_**s**_**' [cm**^**-1**^**]**	**μ**_**a**_**[cm**^**-1**^**]**	**μ**_**s**_**' [cm**^**-1**^**]**	**μ**_**a**_**[cm**^**-1**^**]**		
1	1	Female	30	Cheek	6.1033	0.4132	2.5565	0.0013	Low grade Malignancy	SCC
2	3	Male	60	Cheek	20.1398	0.4108	2.1211	0.0016	Malignant	SCC
3	4	Male	65	Cheek	40.4127	0.2058	3.1488	0.147	Malignant	BCC
4	5	Male	53	Cheek	19.3786	0.4267	3.2852	0.1208	Malignant	BCC
5	6	Male	72	Cheek	13.9541	0.5949	2.0975	0.0016	Malignant	BCC
6	7	Male	42	Cheek	2.0559	0.0603	2.3434	0.0014	Benign	Benign
7	8	Male	46	Cheek	5.787	0.3832	3.5257	0.1475	Low grade Malignancy	SCC
8	9	Male	35	Cheek	5.4704	0.3384	3.6193	0.163	Low grade Malignancy	SCC
9	12	Male	53	Cheek	12.453	0.857	2.9346	0.2001	Malignant	SCC
10	14	Male	48	Cheek	7.963	0.1765	2.9361	0.1451	Malignant	SCC
11	15	Female	47	Cheek	6.5169	0.6583	2.5561	0.1239	Low grade Malignancy	SCC
12	16	Male	43	Cheek	4.3421	0.6342	2.3692	0.0056	Low grade Malignancy	Benign
13	17	Male	38	Cheek	7.5231	0.3541	3.4321	0.0239	Malignant	SCC
14	18	Male	58	Cheek	5.0145	0.8001	3.0189	0.0098	Low grade Malignancy	Benign
15	19	Male	40	Cheek	12.341	0.4271	3.0341	0.0785	Malignant	BCC
16	20	Male	46	Cheek	12.4521	0.8765	3.1452	0.0231	Malignant	BCC
17	22	Female	45	Cheek	10.7432	0.3923	2.9313	0.3817	Malignant	BCC
18	24	Male	51	Cheek	5.8123	0.0969	2.3847	0.0185	Low grade Malignancy	SCC
19	25	Male	35	Cheek	9.1723	0.7453	2.8376	0.2387	Malignant	SCC
20	26	Female	46	Cheek	11.3729	0.813	3.0062	0.1078	Malignant	BCC
21	27	Male	42	Cheek	3.6791	0.2338	2.4312	0.0132	Benign	Benign
22	31	Male	50	Cheek	8.7812	0.3482	2.4932	0.0762	Malignant	BCC
23	32	Male	61	Cheek	6.8231	0.4872	3.0045	0.0392	Low grade Malignancy	SCC
24	33	Male	42	Cheek	7.5237	0.2367	2.4832	0.1289	Malignant	SCC
25	35	Female	39	Cheek	3.3231	0.1293	2.0213	0.0092	Benign	Benign
26	37	Male	38	Cheek	11.2621	0.2761	3.0123	0.0927	Malignant	BCC
27	38	Male	43	Cheek	5.9127	0.3327	2.0128	0.0327	Low grade Malignancy	SCC
28	39	Female	29	Cheek	4.1281	0.0912	2.5321	0.0029	Benign	Benign
29	40	Female	48	Cheek	6.2632	0.2642	2.9327	0.1234	Low grade Malignancy	SCC
30	41	Male	45	Cheek	9.4753	0.3424	3.4842	0.2013	Malignant	BCC
31	43	Male	56	Cheek	12.3473	0.1283	2.8184	0.0281	Malignant	SCC
32	44	Male	47	Cheek	18.4732	0.4328	3.045	0.0294	Malignant	BCC
33	47	Male	37	Cheek	6.0313	0.3591	3.2467	0.0482	Low grade Malignancy	SCC
34	48	Female	46	Cheek	4.2485	0.1439	2.3411	0.0927	Low grade Malignancy	Benign
35	49	Male	33	Cheek	4.4553	0.1943	3.0183	0.0046	Benign	Benign
36	50	Male	58	Cheek	7.2384	0.3872	2.8274	0.06289	Malignant	SCC
The mean value of the reduced scattering coefficient for the healthy cheek skin							100.988			
							2.8052222			

## Abbreviations

OIDR: Oblique Incidence Diffuse Reflectance; SCC: Squamous Cell Carcinomas; BCC: Basal Cell Carcinomas; CCD: Charged Couple Device; MOH: Ministry of Health; NA: Natural Aperture; UV: Ultra Violate; IR: Infra Red.

## Competing interests

The authors declare that they have no competing interests.

## Authors’ contributions

AMA obtained funding for the study, design and implementation the OIDR setup, performing the experimental work, programming the engineering and statistical calculations by Matlab, interpretation of analyzed data, editing, drafting and finalizing the manuscript. MSD supervised the design of OIDR setup, advice the engineering and scientific bases of the research, interpretation of analyzed data, coordinated all the work, drafting and finalizing the manuscript. MKT prepared and selected the patients who are under our study, coordinated the hospital acceptance for the patients testing by our system, follow up the histological diagnosis of the selected patients, advice the dermatological information of the study, drafting and finalizing the manuscript. FAZ advice the histological information of the study, coordinated all work, drafting and finalizing the manuscript. All authors read and approved the final manuscript.
